# Effect of aging on clinical features and metabolic complications of women with polycystic ovary syndrome

**DOI:** 10.1007/s40618-021-01594-5

**Published:** 2021-06-05

**Authors:** P. Falcetta, E. Benelli, A. Molinaro, C. Di Cosmo, B. Bagattini, S. Del Ghianda, G. Salvetti, E. Fiore, E. Pucci, F. Fruzzetti, M. Tonacchera

**Affiliations:** 1 Section of Endocrinology, Department of Clinical and Experimental Medicine, University Hospital of Pisa, Via Paradisa, 2, 56124 Pisa, Italy; 2 Department of Obstetrics and Gynecology, University Hospital of Pisa, Pisa, Italy

**Keywords:** Aging, Polycystic ovary syndrome, Phenotype, Hyperandrogenism, Metabolic syndrome, Insulin resistance

## Abstract

**Purpose:**

To assess the distribution of clinical features and metabolic abnormalities of polycystic ovary syndrome (PCOS) women according to their age.

**Methods:**

Retrospective study on 602 women (mean age 23.9 ± 6.2 years), diagnosed according to International PCOS Network Guidelines criteria as having PCOS in a University-based Hospital. Anthropometric features, hormonal and metabolic parameters were measured and compared between the different age groups (group A ≤ 20 years; group B 21–30 years; group C > 30 years).

**Results:**

Patients in group A were more often hyperandrogenic, while in group C hypertension, dyslipidemia, obesity, impaired fasting glucose, and insulin resistance (IR) were more prevalent. After adjusting for BMI, age correlated positively with sex hormone-binding globulin (SHBG), IR, total- and LDL-cholesterol, and negatively with DHEAS, insulin, and free androgen index (FAI). SHBG was significantly associated with IR and atherogenic dyslipidemia, while FAI levels were linked to hypertension, independently of other factors considered. Furthermore, the regression analysis showed a stronger relationship between BMI and metabolic outcomes, regardless of age.

**Conclusion:**

Polycystic ovarian syndrome (PCOS) phenotype changes with age. Clinical and biochemical hyperandrogenism are a major concern in young PCOS women, while metabolic burden tends to increase with aging. Some of the cardiovascular risk factors are dependent on FAI and SHBG levels, whereas BMI confirms its key role in the genesis of most of the metabolic sequelae in PCOS, independently of age.

## Introduction

Polycystic ovary syndrome (PCOS) is the most common endocrine disorder in women of reproductive age, which is characterized by a complex and heterogeneous clinical presentation that may differ depending on the severity of hormonal and metabolic alterations [1]. The clinical and biochemical presentation of PCOS may change with age, with a relative reduction of its prevalence among older women [2]. Some longitudinal studies suggest an amelioration of PCOS phenotypic characteristics with aging, indicated by an improved cycle regularity [3, 4] and decrease in serum androgen levels during time [5]. Currently available diagnostic criteria [6] take into account the age at presentation of women with PCOS only partially so, since some PCOS cardinal features may become milder, or even normalize, during the lifetime, ignoring the patient's age may result in over- or under-diagnosis of the syndrome. Whereas, some data reported a worsening of metabolic profile of PCOS women with aging [7], others failed to demonstrate it [5, 8]. Hence, the diagnosis of PCOS at different age could implicate different clinical problems and therapeutic strategies. Since most of the available series enrolled a small number of subjects with a limited number of variables assessed, the present study aims to enrich the current knowledge by evaluating the effect of aging on clinical, hormonal, and metabolic features of a large number of women with PCOS.

## Materials and methods

### Subjects enrolled

In this retrospective study, 602 consecutive PCOS patients, recruited at the Department of Endocrinology of Pisa from February 1, 2014 to November 30, 2019 were included. The study was conducted in accordance with the Helsinki declaration of 1975, as revised in 1983. Ethical approval was waived by the local Ethics Committee of University of Pisa in view of the retrospective nature of the study and all the procedures being performed were part of the routine care. Data were pseudonymized from patients’ clinical records and anonymously analyzed. All participants gave their informed consent to participate in the study. The gynecological age of all patients was > 2 years. Subjects were categorized according to their age: group A, ≤ 20 years; group B, 21–30 years; group C, > 30 years. Anamnestic data about menstrual cycle irregularities were collected, and clinical exam, including the evaluation of any signs of hyperandrogenism such as acne, alopecia, hirsutism, was performed. Clinical evaluation included measurement of weight and height, body mass index [BMI, weight (kg)/height (m)^2^], systolic (SBP) and diastolic (DBP) arterial blood pressure, and heart rate (HR). BMI levels were categorized as follows: normal weight, BMI 18–24.9 kg/m^2^; overweight, BMI 25–29.9 kg/m^2^; obesity, BMI ≥ 30 kg/m^2^. In all patients, fasting blood samples were collected during the follicular phase of a spontaneous or induced menstrual cycle. Plasma levels of luteinizing hormone (LH), follicle-stimulating hormone (FSH), total testosterone (TT), delta 4-androstenedione (A4), sex hormone-binding globulin (SHBG), dehydroepiandrosterone sulfate (DHEAS), fasting insulin and plasma glucose (FPG) were measured. Eventually, LH/FSH ratio as a surrogate marker of gonadotropic axis alteration and DHEAS/TT ratio as a marker of androgen secretion source were calculated. Ultrasonography evaluation of ovarian morphology was carried out in day 2–4 of a cycle by a single experienced operator (FF) using a transvaginal or an abdominal probe (Hitachi 3.5-MHz and 5 MHz).

Hypertension was defined according to 2018 ESC/ESH criteria [9]. Diabetes mellitus, impaired fasting glucose (IFG), and impaired glucose tolerance (IGT) were defined using American Diabetes Association criteria [10]. Dyslipidemia was defined in the presence of one or more of these criteria: (i) total cholesterol levels ≥ 200 mg/dL (5.17 mmol/l), combined with LDL cholesterol > 150 mg/dl (3.88 mmol/l) or HDL cholesterol ≤ 50 mg/dl (1.3 mmol/l) for females and ≤ 40 mg/dl (1 mmol/l) for males; (ii) triglycerides ≥ 150 mg/dl (1.69 mmol/l); (iii) use of lipid-lowering medications. Atherogenic dyslipidemia was defined in presence of both low HDL-C (< 50 mg/dl) and high TG (> 150 mg/dl).

Patients with adrenogenital syndrome or congenital adrenal hyperplasia (CAH), ovarian hypertecosis, androgenic-secreting ovarian or adrenal tumors, Cushing's syndrome, idiopathic hyperandrogenism, idiopathic hirsutism, hyperprolactinemia, were excluded. The concentration of 17–OH–progesterone (17–OH–P) was measured. When basal 17–OH–P levels were > 2 ng/ml, an ACTH test was performed to exclude the presence of CAH. 17–OH–P was measured before and after 30 min from the injection of 0.25 mg of synthetic ACTH (tetracosactide). Patients with 17–OH–P values > 10 ng/ml after ACTH test were excluded from the study. Cushing’s syndrome was excluded by the overnight 1-mg dexamethasone suppression test (1 mg-DST); cortisol values ≤ 1.8 mcg/dl were considered normal, thus included in the study. Patients with idiopathic hyperandrogenism (clinical and/or biochemical hyperandrogenism, without oligo-anovulation and PCOM at pelvic ultrasound) or idiopathic hirsutism (clinical hyperandrogenism without biochemical hyperandrogenism, oligo-anovulation, PCOM) were excluded. Patients with ovarian or adrenal tumors diagnosed according to laboratory and imaging data and patients with hyperprolactinemia (serum PRL levels above the normal range of the assay used at our institution, i.e. 25–30 ng/ml) were excluded. None of the patients had taken medication known to interfere with all the biochemical determinations performed in the six months prior to the study enrollment.

### PCOS diagnostic criteria

For adult patients, the diagnosis of PCOS was made following the International PCOS Network Guidelines [6], thus including in the study the patients who presented at least two among:

1. Clinical and/or biochemical hyperandrogenism. Clinical hyperandrogenism was defined as the presence of acne and/or hirsutism. A Ferriman–Gallwey (FG) score equal or greater than 8 was indicative of hirsutism [11]. Biochemical hyperandrogenism was diagnosed when at least one of the following was present in two different determinations: TT ≥ 0.8 μg/l; free androgen index (FAI) > 7.1%.
2. Irregular menstrual cycles, defined as having less than 8 menstrual cycles/year, absence of 3–6 mestrual cycles/year, or intermenstrual interval > 35 days.
3. Polycystic ovarian morphology (PCOM) at pelvic ultrasound scan, defined in presence of at least one ovary with ≥ 20 follicles measuring 2 and 9 mm, regardless of their arrangement and/or ovarian volume > 10 ml or cm^3^.

All the adolescents were diagnosed as having PCOS when clinical (severe acne or hirsutism) or biochemical evidence of hyperandrogenism was present together with oligo-amenorrhea, the latter for at least 2 consecutive years. An ovarian volume > 10 ml or cm^3^ was considered enlarged, while follicle counts was not used to define PCOM [12]. Nonetheless, PCOM was not used for the diagnosis of PCOS in this age-group.

### Biochemical determinations

A4 levels were determined using a radioimmunoassay (Diasource Europe S.A., Nivelles, Belgium; range 0.1–11 ng/ml). The intra-assay and the interassay coefficient of variation (CV) were 3.2–4.5% and 5.9–9.0%, respectively. TT concentrations were determined using a competitive immunoassay (Johnson & Johnson S. p. A-Ortho Clinical Inc.; range 0.06–0.77 ng/ml). The intra-assay and interassay CV were 2.3–3.1% and 4.9–7.0%, respectively. The concentrations of DHEAS were determined by using a radioimmunoassay (Orion Diagnostica, Espoo, Finland). The intra-assay and the inter-assay CV were 3.5%–6.5% and 4.0%–8.1%, respectively. SHBG levels were detected by immunoassay (Access Immunoassay System, SHBG, Beckman Coulter, Brea, CA, USA; 13–89.5 nmol/l). The intra-assay and interassay CV were < 7%. Insulin was determined by an immunoradiometric assay (DiaSorin S. p. A., Vercelli, Italy; range: 4–16 µIU/ml). The intra-assay and interassay CV were 2.1–2.6% and 2.9–4.7%, respectively. Glucose levels were assessed by enzymatic methods (Roche Diagnostics, Basel, Switzerland). The free androgen index (FAI) was calculated with the formula: FAI = *T* (nmol/l) × 100/SHBG (nmol/l) [13].

Insulin resistance was evaluated by the Quantitative Insulin Sensitivity Check Index (QUICKI) calculated according to the formula: 1/[logFasting Insulin (**μ**U/l) + fasting glucose (nmol/l)] [14]. Women with a QUICKI value < 0.357 were considered insulin-resistant [15]. Lipid profile was evaluated measuring serum levels of total cholesterol (TC), high-density lipoprotein cholesterol (HDL-C), low-density lipoprotein cholesterol (LDL-C), triglycerides (TG). SI conversion factors: fasting plasma glucose × 0.0555 nmol/l; fasting insulin × 6.00 pmol/l; testosterone × 3.467 nmol/l; total cholesterol/HDL-C/LDL-C × 0.02586 nmol/l; Tg × 0.0113 nmol/l; DHEA-S × 0.02714; androstenedione × 0.033.

### Statistical analysis

Variables were tested for normal distribution with the Shapiro–Wilk test and expressed as mean ± SD, or median (interquartile range [IQR]), as appropriate. Analysis of variance (ANOVA) with Scheffe post-hoc test for multiple comparisons or Kruskal–Wallis test was used to determine group differences in parametric and non-parametric variables, respectively. Categorical variables were compared by the Chi-squared test or Fisher’s exact tests. The Mantel–Haenszel test of trend was used to assess the linear association between age-group and categorical variables. The Pearson’s correlation coefficient was used to quantify the association between normally distributed variables, while the Spearman correlation coefficient was used for non-parametric variables. Univariate and multivariate logistic regression analysis was performed to assess the association between anthropometric and biochemical features and metabolic outcomes. Due to multiple tests, we applied the conservative Bonferroni–Holms correction. Statistical significance was set at two-tailed *P* < 0.05. All statistical analyses were processed using SPSS (IBM SPSS statistics, Version 25).

## Results

### General characteristics

A total of 602 patients were recruited (mean age 23.9 ± 6.2 years; IQR, 19–28; range 14–53). The majority (*n* = 301; 50%) were in the group B (21–30 years), while 205 (34.1%) were in the group A (≤ 20 years) and 96 (15.9%) were in the group C (> 30 years). Clinical, anthropometric, biochemical, and hormonal characteristics of PCOS women, stratified by age, are summarized in Table 1. The three groups were comparable for the LH/FSH ratio. Estradiol was higher in patients in the group C as compared to those in group A and B. TT levels were highest in the group A, in which concentrations of TT were significantly higher than group C and non-different compared to those in group B. Similarly, SHBG levels were significantly lower and FAI significantly higher in group A than group C. DHEAS levels were significantly higher in group C as compared with group A and B. DHEAS/TT ratio was significantly lower in group C than in group A and B. The levels of A4 tended to decrease along with age, with the highest concentrations observed in group A, progressively reducing in group B and C. BMI was significantly higher in group C than in group A and B. Similarly, SBP and DBP were higher in patients of group C as compared to those in group A and B. Regarding the glycemic indexes, FPG was higher in group C than group A and B, while QUICKI and fasting insulin were higher in group A and C compared to patients in group B. When the lipid profile was considered, TC, LDL-C, and triglycerides were significantly higher in group B and C than in group A, while HDL-C was lower in group A than in group B.

**Table 1:** Clinical and biochemical characteristics of PCOS women according to the age at diagnosis

	Group A (≤ 20 years)	Group B (21–30 years)	Group C (> 30 years)	*P*
*N*, %	205 (34.1)	301 (50)	96 (15.9)	
Age, years	17.6 ± 1.9	24.8 ± 2.7	34.3 ± 3.7	< 0.0001
BMI, kg/m^2^	28.5 ± 6.2	28.1 ± 7.9	31.7 ± 8.7^a,b^	< 0.0001
FG	14 (8–20)	13 (8–19)	12 (8–16.7)	0.219
SBP, mmHg	120.2 ± 10.6	118.7 ± 10.4	125.5 ± 14.8^a,b^	< 0.0001
DBP, mmHg	76.6 ± 7.5	76.9 ± 8.3	80.5 ± 9.9^a,b^	0.004
FPG, mg/dl	83.4 ± 15.7	83 ± 9.1	89.6 ± 16.7^a,b^	< 0.0001
QUICKI	0.34 ± 0.035^b^	0.36 ± 0.035	0.34 ± 0.037^b^	< 0.0001
Insulin, μIU/ml	14.3 ± 12.5^b^	10.4 ± 9.1	13 ± 9.0^b^	< 0.0001
TC, mg/dl	170.4 ± 34.8^b,c^	179.6 ± 29.1^a,c^	194.4 ± 35.4^a,b^	< 0.0001
HDL-C, mg/dl	57 ± 14.2^b^	60.9 ± 16.2^a^	58.6 ± 15.0	0.033
LDL-C, mg/dl	105 ± 29.5^c^	110.2 ± 26.3^c^	122.7 ± 31.1^a,b^	< 0.0001
TG, mg/dl	74 (57–102)	71.5 (55–99.2)	89 (66–140)^a,b^	0.001
LH/FSH	1.9 ± 1.3	1.8 ± 1.1	1.7 ± 1.1	0.559
E2, pg/ml	60.9 ± 38.4	67.9 ± 48.1	76.9 ± 48.8^a^	0.010
TT, ng/ml	0.8 (0.6–0.9)	0.7 (0.6–0.9)	0.6 (0.5–0.8)^a,b^	0.003
SHBG, nmol/l	22.6 (16.7–35.4)	31 (21.9–46.2)^a^	31.3 (21.4–51.4)^a^	< 0.0001
FAI, %	11.2 (7.2–17.4)	8.3 (4.9–13)^a^	7 (4.4–10.8)^a^	< 0.0001
DHEAS, μg/dl	263 (191.4–339.5)	247 (183–323)	189 (134.1–255.9)^a,b^	< 0.0001
A4, ng/ml	3.2 (2.3–4.3)	3 (2.4–4.2)	2.8 (2.1–3.6)	0.050
DHEAS/TT	347.7 (249.6–471)	340.5 (250.2–448.9)	291.4 (203–387.6)^a,b^	0.002

*BMI* body mass index, *FG* Ferriman–Gallwey score, *SBP* systolic blood pressure, *DBP* diastolic blood pressure, *FPG* fasting plasma glucose, *TC* total cholesterol, *TG* triglycerides, *E2* estradiol, *TT* total testosterone, *SHBG* sex hormone-binding globulin, *FAI* free androgen index, *A4* 4-androstenedione, *DHEAS* dehydroepiandrosterone sulfate

Scheffe post-hoc test: ^a^*P* < 0.05 vs group A; ^b^*P* < 0.05 vs group B; ^c^*P* < 0.05 vs group C

### Age-related differences in PCOS clinical features, biochemical parameters, and metabolic complications

**Fig. 1:**
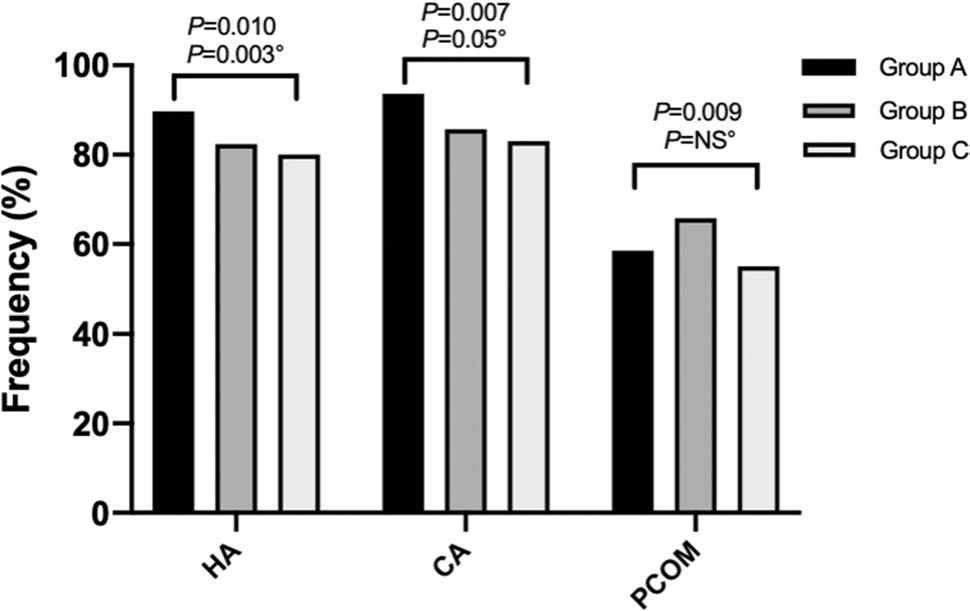
Distribution of clinical features in PCOS women according to the age at diagnosis. *CA* chronic anovulation, *HA* hyperandrogenism, *PCOM* polycystic ovarian morphology. *P* values: Pearson’s Chi-squared or Fisher’s exact test, when appropriate. °Mantel–Haenszel test of trend

**Fig. 2:**
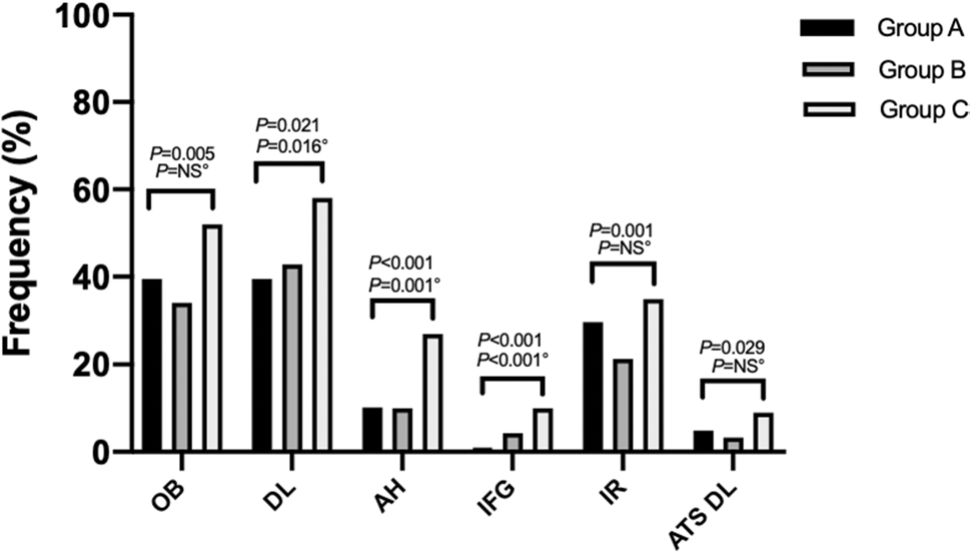
Distribution of metabolic alterations in PCOS women according to the age at diagnosis. *AH* arterial hypertension, *ATS DL* atherogenic dyslipidemia, *DL* dyslipidemia, *IFG* impaired fasting glucose, *IR* insulin resistance. *P* values: Pearson’s Chi-squared or Fisher’s exact test, when appropriate. °Mantel–Haenszel test of trend

The prevalence of clinical features and metabolic abnormalities among different age groups is shown in Figs. 1 and 2. Hyperandrogenism was more frequently encountered in group A (89.7%) than group B and C (82.4 and 80%, respectively; *P* = 0.010), and its prevalence tended to decrease with aging (M–H *P* = 0.003). The prevalence of chronic anovulation was significantly different between groups (*P* = 0.007), being highest in younger patients (group A, 93.6%) and lowest in group C (83%). The PCOM was more frequently encountered in patients in group B (65.8%) than those in group A and C (58.5 and 55%, respectively). Obesity was more commonly found in group C than in group A and B (52 vs 39.5 and 34.1%, respectively; *P* = 0.005). The prevalence of dyslipidemia differed between age groups (*P* = 0.016), with a tendency to increase with advancing age (M–H *P* = 0.021); the atherogenic lipid pattern was significantly higher in group C (9%) compared to group A (4.9%) and B (3.3%; *P* = 0.029). Hypertension was threefold more frequent in group C than group A and B (27 vs 10.2 and 10%, respectively; *P* < 0.0001) while the prevalence of IR and IFG was significantly higher in group C (35% and 10%, respectively) than group A (29.7 and 1%) and B (21.3 and 4.3%; all *P* < 0.05) Unadjusted and BMI-adjusted correlations were performed to assess the relationship between age and anthropometric and biochemical parameters. Results are shown in Table 2. Age was positively correlated with SHBG concentrations, TC, HDL-C, and LDL-C, while it was inversely related to TT, FAI, DHEAS, insulin, and DHEAS/TT ratio. Since BMI differed significantly between age groups, a partial-correlation analysis adjusted for BMI was performed. The positive relationship between age and SHBG, TC, HDL-C, LDL-C, and the negative relationship with DHEAS, insulin, FAI, and QUICKI was kept independently of BMI values. Furthermore, SHBG correlated inversely with fasting insulin (*r*: − 0.417; *P* < 0.0001), BMI (*r*: − 0.546; *P* < 0.0001), TT (*r*: − 0.123; *P* = 0.004), and TG (*r*: − 0.298; *P* < 0.0001), while it was positively correlated with HDL-C (*r*: 0.486; *P* < 0.0001), and QUICKI (*r*: 0.416; *P* < 0.0001). BMI was significantly associated with insulin levels (*r*: 0.509; *P* < 0.0001). When adjusting for BMI levels, the association between SHBG and fasting insulin levels was no more statistically significant (*r*: − 0.060; *P* = 0.512) while the other significant associations were confirmed (data not shown).

**Table 2:** Unadjusted and BMI-adjusted correlations between age and different biochemical and hormonal parameters in PCOS women

	Unadjusted			Partial correlations^§^		
	*R*	*R*^2^	*P*	*R*	*R*^2^	*P*
SHBG	0.238	0.056	< 0.0001	0.390	0.152	< 0.0001
TT	− 0.106	0.011	0.010	0.025	0.000	0.619
DHEAS	− 0.184	0.033	< 0.0001	− 0.193	0.040	< 0.0001
TC	0.265	0.070	< 0.0001	0.264	0.070	< 0.0001
HDL-C	0.103	0.011	0.020	0.194	0.040	< 0.0001
LDL-C	0.199	0.040	< 0.0001	0.182	0.033	< 0.0001
DHEAS/TT	− 0.138	0.020	0.001	− 0.055	0.003	0.281
Insulin	− 0.118	0.014	0.005	− 0.170	0.030	0.001
QUICKI	0.061	0.004	0.231	0.174	0.030	0.001
FAI	− 0.231	0.053	< 0.0001	− 0.251	0.063	< 0.0001

*DHEAS* dehydroepiandrosterone sulfate, *FAI* free androgen index, *SHBG* sex hormone-binding globulin, *TT* total testosterone, *TC* total cholesterol, *QUICKI* quantitative insulin sensitivity check index

^§^Values are adjusted for BMI

### Predictors of metabolic abnormalities in PCOS women

To evaluate the association between age, clinical features, biochemical parameters, and metabolic abnormalities, a uni- and multivariable logistic regression analysis was performed. Results are reported in Table 3. The univariate analysis showed that age, BMI, SHBG and FAI levels were associated with IR while TT was not. The final model using a backward conditional approach included age class, BMI, and SHBG as significant predictors of IR. Briefly, patients in the age group A and B were less likely to be IR (group A: OR 0.56; 95% CI 0.24–1.31; group B: OR 0.44; 95% CI 0.20–0.95) compared to older patients (group C), with an independent contribution of BMI (OR 1.11; 95%CI 1.07–1.16) and SHBG (OR 0.96; 95% CI 0.94–0.98). After adjusting for confounders, dyslipidemia was associated only with BMI (OR 1.11; 95% CI 1.08–1.15). Regarding the atherogenic dyslipidemic pattern (high triglycerides and low HDL-C), in the multivariable analysis, it was associated with BMI (OR 1.12; 95% CI 1.06–1.18) and inversely with SHBG values (OR 0.96; 95% CI 0.92–0.99; *P* = 0.035). Finally, patients in the lower age groups (group A and B) were less likely to be hypertensive compared to those in the group C (OR 0.38; 95% CI 0.17–0.85 and OR 0.40; 95% CI 0.20–0.80 for Group A and B, respectively). At the multivariable analysis, BMI (OR 1.13; 95% CI 1.09–1.18) and FAI (OR 1.04; 95% CI 1.00–1.08) were also independently associated with hypertension.

**Table 3:** Logistic regression analysis for predictors of metabolic complications in PCOS women

	IR				Dyslipidemia				Atherogenic dyslipidemia				Hypertension			
	Univariate		Multivariate		Univariate		Multivariate		Univariate		Multivariate		Univariate		Multivariate	
	OR (95% CI)	*P* value	OR (95% CI)	*P* value	OR (95% CI)	*P* value	OR (95% CI)	*P* value	OR (95% CI)	*P* value	OR (95% CI)	*P* value	OR (95% CI)	*P* value	OR (95% CI)	*P* value
Age class																
< 20	0.76 (0.44–1.30)	0.323	0.56 (0.24–1.31)	0.182	0.48 (0.28–0.83)	0.008	–	–	0.47 (0.18–1.21)	0.117	–	–	0.30 (0.16–0.58)	< 0.0001	0.38 (0.17–0.85)	0.019
21–30	0.42 (0.25–0.71)	0.001	0.44 (0.20–0.95)	0.038	0.50 (0.30–0.84)	0.008	–	–	0.30 (0.12–0.76)	0.011	–	–	0.28 (0.15–0.50)	< 0.0001	0.40 (0.20–0.80)	0.010
> 30	Ref		Ref		Ref				Ref				Ref			
BMI	1.16 (1.12–1.19)	< 0.0001	1.11 (1.07–1.16)	< 0.0001	1.11 (1.08–1.14)	< 0.0001	1.11 (1.08–1.15)	< 0.0001	1.14 (1.09–1.19)	< 0.0001	1.12 (1.06–1.18)	< 0.0001	1.15 (1.11–1.19)	< 0.0001	1.13 (1.09–1.18)	< 0.0001
SHBG	0.94 (0.92–0.95)	< 0.0001	0.96 (0.94–0.98)	0.001	0.99 (0.98–0.99)	< 0.0001	–	–	0.93 (0.89–0.97)	< 0.0001	0.96 (0.92–0.99)	0.035	0.97 (0.95–0.98)	< 0.0001	–	–
FAI	1.06 (1.04–1.09)	< 0.0001	–	–	1.04 (1.02–1.07)	< 0.0001	–	–	1.04 (1.00–1.07)	0.024	–	–	1.04 (1.01–1.06)	0.001	1.04 (1.00–1.08)	0.034
TT	1.24 (0.82–1.86)	0.302	–	–	1.45 (0.89–2.36)	0.130	–	–	0.78 (0.21–2.95)	0.720	–	–	1.10 (0.66–1.82)	0.719	1.23 (0.82–1.91)	0.419

*BMI* body mass index, *FAI* free androgen index, *IR* insulin resistance, *SHBG* sex hormone-binding globulin, *TT* total testosterone

## Discussion

In this large cross sectional study performed on more than 600 women, we observed that clinical and biochemical features of PCOS change with aging. Ovarian dysfunction and hyperandrogenism are major concerns in younger women, while metabolic disturbances are predominant as PCOS women get older. However, regardless of age, metabolic abnormalities are frequently encountered in PCOS women. In our cohort and as well as previously reported by others [3, 8, 16–18], menstrual pattern improved over time. These results were strengthened by the exclusion of women with a gynecological age < 2 years, since the physiological maturation of the reproductive system in the first years after menarche is frequently characterized by menstrual irregularities. The improvement in cycle pattern may be explained by the natural decline of the number of ovarian follicles and ovarian steroid secretion capacity with age, the latter partially resolving before menopause in women with PCOS [8, 18]. In keeping with this, we found that DHEAS, TT, and FAI levels were negatively associated with age. Despite controversies, most data support the knowledge that as women grow older, there is a significant decrease in androgen production [4]. The mechanisms are largely attributed to a decreased theca cell number and function in PCOS over time [19]. Differently from other authors’ findings [8] and in accordance to what reported in a recently published large Brazilian study on 796 PCOS women [20], we observed a significant decrease in circulating androgens in PCOS women already at the age of 20 compared to those with younger age. A similar relationship between age and hyperandrogenism was also reported by Livadas et al*.* in their large cross sectional study performed on 1345 women with PCOS [21]. The inconsistency of results may be explained by the higher proportion of PCOS women < 20 years enrolled in this study as compared to previous series. The progressive decline of FAI with aging may be dependent on both the reduced androgen production and parallel SHBG increase. In keeping with previous studies and being note the suppressive effect of insulin on hepatic production of SHBG [22], we found that SHBG was inversely related to insulin levels. However, when adjusting for BMI, this relationship was no more significant, probably due to the stronger impact of body mass on both SHBG and insulin production. Furthermore, age was found as a significant predictor for insulin levels in PCOS women. This finding is in accordance with studies reporting a relative insulin secretory defect [22], incretin insensitivity of beta-cells [23], and decrease of muscle-related insulin sensitivity and expression of skeletal muscle glucose transporter 4 (SGLT-4) [24] with aging. However, studies using the hyperinsulinemic–euglycemic clamp for the assessment of IR, have shown that adiposity and physical inactivity are the primary determinants of the age-related insulin resistance rather than chronological age [25]. Supporting these results, Livadas and colleagues reported that IR tends to decline with age in non-obese PCOS, while this phenomenon is not observed in obese patients [21]. In contrast, we found that the association between IR and age was retained regardless of body mass index, suggesting that the association between obesity and metabolic disturbances in women with PCOS becomes clear later in life. The apparent inconsistency of results may be explained by the higher BMI of patients included in the present study, especially those in group C, in which body mass and age were greatest. These data might explain—per se—the higher frequency of glucose abnormalities found in older PCOS women. Interestingly, we found that insulin resistance displayed a U-shaped distribution, being highest in those ≤ 20 and > 30 years. These results are in accordance to what reported by others [16, 26] and could be directly related to the higher BMI found in patients in group A and C as compared to those in Group B. Younger PCOS women were found to be not only more hyperandrogenic, but also metabolically unhealthy. Metabolic abnormalities were highly dependent on BMI, supporting the conclusion that dietary and lifestyle interventions are crucial for preventing progression to poorer reproductive and metabolic outcomes in PCOS women. The tight association between cardiometabolic risk factors and adipose-dependent IR has been recently reported by Mu et al. [27]. The mechanisms underlying adipose insulin resistance remain unclear, however, it seems to be partly mediated by chronic low-grade inflammation, not only in PCOS [28] but also in obese non-PCOS individuals [29]. Nevertheless, several IR signaling pathways are upregulated by inflammation [30]. The logistic regression analysis showed that SHBG was associated with insulin-resistance and atherogenic dyslipidemia, independently of BMI. Several studies reported a significant positive correlation between SHBG and HDL cholesterol in healthy [31, 32] as well as PCOS women [33], even after adjusting for multiple confounders. We also found that hyperandrogenism, expressed as FAI, was associated with hypertension regardless of other factors considered. In contrast, TT was not associated with any of the metabolic outcomes evaluated, underlining the higher accuracy of FAI or bioavailable testosterone for the assessment of androgen status and its metabolic implications in PCOS women [34]. A strong independent association between SHBG, FAI, and cardiovascular disease risk factors (i.e. obesity, insulin, glucose, and HDL levels, DBP, inflammatory markers) has been previously reported in peri- and premenopausal women of the large SWAN (Study of Women across the Nation) study [35]. Similarly, Chen and colleagues, in a cross sectional study, including 151 young PCOS women, found a significant positive relationship between FAI, TT and SBP/DBP levels, independently of insulin resistance, obesity, and dyslipidemia [36]. The mechanisms accounting for this association are not fully clarified. However, there is evidence that androgens may upregulate the proximal tubule renin–angiotensin system and increase the resorptive volume rate, thereby increasing extracellular volume and blood pressure [37]. Endothelial dysfunction was shown to be associated with androgens levels in postmenopausal women [38]; more recently, epicardial fat and carotid intima media thickness have been found to be higher in women with PCOS and idiopathic hirsutism compared to healthy controls [39]. We did not find any association between hyperandrogenism and IR or lipid abnormalities, though a recent meta-analysis have shown that hyperandrogenism may play a role between PCOS and metabolic syndrome features [40]. All these findings suggest that the characteristic hyperandrogenism occurring in women of reproductive age with PCOS might contribute to the higher cardiovascular risk observed in this population [41]. Certain limitations of this study must be considered. The cross sectional design is limited to reveal possible associations in a determined moment and does not disclose a cause–effect relationship. However, to date only a few small longitudinal studies investigating the effect of age on PCOS features have been performed [3–5, 8, 17]. In consideration of this critical point, the availability of data collected in a large sample of PCOS women may give us the opportunity to arise some conclusions. The absence of a control group may be considered another limitation of this study. However, the main scope of the research was to evaluate the metabolic features of intra-PCOS group according to age and not to directly compare PCOS women with healthy controls. Moreover, being a retrospective series, several important anthropometric and biochemical variables such as waist circumference, waist–hip ratio, and serum AMH were not available. Finally, this is a university center-based study; therefore, we cannot exclude the presence of a referral bias, so our results may not be expandable in different populations.

In conclusion, the PCOS phenotype changes with age. Clinical and biochemical hyperandrogenism are a major concern in young PCOS women, while metabolic burden tends to increase with aging. Some of the cardiovascular risk factors investigated are dependent on FAI and SHBG levels, whereas, BMI confirms its key role in the genesis of most of the metabolic sequelae in PCOS, independently of age. Therefore, the prevention and management of hyperandrogenism and weight excess need to be addressed early to avoid the development of metabolic disturbances in PCOS women.

## Data Availability

The data that support the findings of this study are available from the corresponding author upon reasonable request.
